# Robust consensus tracking control of multiple mechanical systems under fixed and switching interaction topologies

**DOI:** 10.1371/journal.pone.0178330

**Published:** 2017-05-25

**Authors:** Jianhui Liu, Bin Zhang

**Affiliations:** 1 School of Automation Science and Electrical Engineering, Beihang University (BUAA), Beijing 100876, P.R. China; 2 School of Automation, Beijing University of Posts and Telecommunications (BUPT), Beijing 100876, P.R. China; Chongqing University, CHINA

## Abstract

Consensus tracking problems for multiple mechanical systems are considered in this paper, where information communications are limited between individuals and the desired trajectory is available to only a subset of the mechanical systems. A distributed tracking algorithm based on computed torque approach is proposed in the fixed interaction topology case, in which a robust feedback term is developed for each agent to estimate the external disturbances and the unknown agent dynamics. Then the result is extended to address the case under switching interaction topologies by using Lyapunov approaches and sufficient conditions are given. Two examples and numerical simulations are presented to validate the effectiveness of the proposed robust tracking method.

## Introduction

Multi-agent system has emerged as an active area of research, and drawn attention of scholars from a varieties of disciplines in the past decades. This trend is triggered by the promising applications of multi-agent system in fields like disaster rescuing, industry assembly lines, surveillance, etc. Each agent in the multi-agent system has limited task abilities. However, through interactions with each other, they can work as a team and accomplish cooperative behaviors such as consensus [1, 2], flocking [3], formation [4, 5], and state estimation [6, 7]. Among the studies of these cooperative behaviors, consensus behavior is the most fundamental one [8].

The basic issue of consensus control in multi-agent system is to design a distributed consensus law such that all the agents could be driven to an agreement. In recent years, many consensus control approaches have been proposed for multi-agent systems with different interaction topologies and dynamic models. In the early literature [9], graph theory was used to represent the interaction topologies, and as a result, the relationship between system stability and Laplacian eigenvalues was precisely revealed. In [10], directed graphs were used to represent the interaction topology, and results under dynamically changing interaction topologies were derived. Other representative literatures are [11–13], to name a few. [11] studied finite-time consensus problems for first-order integrators. [12] extended the problems to systems with uncertain dynamics based on *H*_∞_ control theory. [13] considered the constrained consensus problem with a global optimization function.

In addition, some results extended the consensus problem to a more general consensus tracking problem where the agents track a time-varying trajectory instead of a static equilibrium. In [14], a robust adaptive control algorithm was proposed for uncertain nonlinear systems, where the reference trajectory was known to all the following systems. In [15], the problem was solved under the condition that the reference trajectory was available only to a few agents. In [16], finite-time tracking control of multi-agent systems was considered with a sliding-mode approach. In [17], distributed observers were established to estimate unavailable system states. The results in [18] addressed consensus problems with the assumption that the second-order derivatives of the reference signals conform to some given policy known to the system.

Most literatures dealing with consensus tracking problem are presented under the assumption that the dynamics of the agents are linear and certain. However, almost all the physical plants exhibit some kinds of nonlinearities, and external disturbances are inevitable in their dynamical processes. Several works attempt to address the tracking problem with uncertainties, but assumptions of these results are too conservative to achieve in practical situations. Motivated by the desire to achieve practical results with only necessary constraints on mechanical systems and reference trajectory, we try to address the robust consensus tracking problem in this paper. We aim to design a controller such that a group of mechanical systems under both fixed and switching topologies could maintain a satisfactory collective performance in the presence of uncertainties or external disturbances. In the fixed topology case, a distributed robust control law is devised based on the computed torque approach and algebraic graph theory. We further extend the results to the switching topology case. Sufficient conditions are given, under which the states of the agents could converge to a neighbourhood of the origin. Two numerical simulations are conducted to validate the effectiveness of our results.

The remainder of this paper is organized as follows. First, we introduce the problem formation and the relevant notations. Then, the robust tracking control under fixed and switching topologies is discussed. In the Simulation Section, two numerical simulations are conducted to validate the effectiveness of the proposed method. At last, some discussions are made to conclude this paper.

## Problem statement

For a group of *n* mechanical systems, the dynamic model of the *i*th system is formulated by Euler-Lagrange equation [19, 20]

$$\begin{array}{c} M_{i}\left(q_{i}\right)\ddot{q_{i}}+C_{i}\left(q_{i},\dot{q_{i}}\right)\dot{q_{i}}+G_{i}\left(q_{i}\right)+f_{i}\left(\dot{q_{i}}\right)+u_{i}\left(t\right)=\tau_{i} \end{array}$$ (1)

where $q_{i}\in R^{m}$ is the state of the *i*th system, $M_{i}\left(q_{i}\right)\in R^{m\times m}$ is the symmetric inertia matrix, $C_{i}\left(q_{i},\dot{q_{i}}\right)\in R^{m\times m}$ is the matrix representing the centrifugal and Coriolis terms, $G_{i}\left(q_{i}\right)\in R^{m}$ is the vector of gravity terms, $f_{i}\left(\dot{q_{i}}\right)\in R^{m}$ is the frictional term, $u_{i}\left(t\right)\in R^{m}$ denotes the bounded external disturbance and $\tau_{i}\in R^{m}$ represents the control input vector. An assumption of the mechanical system equation described by Eq (1) is given as follows [20, 21]:

**Assumption 1**. The symmetric inertial matrix *M*_*i*_(*q*_*i*_), i∈{1,…,n}≜N, is positive definite, which satisfies:

$$\begin{array}{c} \lambda_{m}{∥x∥}^{2}⩽x^{T}M_{i}\left(q_{i}\right)x⩽\lambda_{M}{∥x∥}^{2},\;\forall q_{i},x\in R^{m} \end{array}$$ (2)

where

$$\begin{array}{ccc} \lambda_{m} & ≜ & \min_{\forall i\in N}\min_{\forall q_{i}\in R^{m}}\lambda_{\min}\left(M_{i}\left(q_{i}\right)\right)\\ \lambda_{M} & ≜ & \max_{\forall i\in N}\max_{\forall q_{i}\in R^{m}}\lambda_{\max}\left(M_{i}\left(q_{i}\right)\right) \end{array}$$

In this paper, it is assumed that the information interchanges are bi-directional between the *n* agents through wireless networks or other sensors. Undirected graphs will be used throughout the paper to model the bi-directional interaction topologies among agents. Some basic knowledge and conventional notations in algebraic graph theory are given as follows. Let G(ν,ε) be an undirected graph with *n* nodes *ν* = {*ν*_1_, *ν*_2_, …, *ν*_*n*_} and the set of edges *ε* ⊆ *ν* × *ν*. The adjacency matrix *A* = [*a*_*ij*_] is a symmetric matrix defined as *a*_*ii*_ = 0 and *a*_*ij*_ > 0 ⇔ (*ν*_*i*_, *ν*_*j*_) ∈ *ε*. The Laplacian matrix of graph G(ν,ε) is defined as *L* = *D* − *A*, where *D* = diag{*d*_1_, *d*_2_, …, *d*_*n*_} is a diagonal matrix with diagonal entries $d_{i}=\sum_{j=1}^{n}a_{ij}$ for *i* = 1, 2, …, *n*. The set of neighbors of node *ν*_*i*_ is denoted by $N_{i}=\left\{\nu_{j}\in \nu |\left(\nu_{i},\nu_{j}\right)\in \varepsilon \right\}$. If there is a path between any two nodes of the graph G(ν,ε), then G(ν,ε) is said to be connected. Suppose *ζ* is a nonempty subset of nodes *ν*, then G(ν,ε⋂(ζ×ζ)) is termed as an induced subgraph by *ζ*. A component of a graph G(ν,ε) is defined as a maximal induced subgraph of G(ν,ε) that is strongly connected. To characterize the variable interconnection topology, a piecewise-constant switching signal function σ(t):[0,∞)→{1,…,M}≜M is defined, where $M\in Z^{+}$ is the total count of possible interconnection graphs.

The reference signals are denoted as $q_{d},\dot{q_{d}}$ and $\ddot{q_{d}}$ respectively. In this paper, $q_{d},\dot{q_{d}}$ and $\ddot{q_{d}}$ are only accessible to a subset of the *n* agents. The accesses of the agents to the trajectories are represented by a diagonal matrix $C=\text{diag}\left\{c_{1},\ldots ,c_{n}\right\}\in R^{n\times n}$, where

$$\begin{array}{c} c_{i}=\{\begin{array}{cc} 1, & \text{if}\;\text{trajectory}\;\text{signals}\;\text{are}\;\text{available}\;\text{to}\;\text{agent}\;i\text{;}\\ 0, & \text{if}\;\text{trajectory}\;\text{signals}\;\text{not}\;\text{available}\;\text{to}\;\text{agent}\;i\text{.} \end{array} \end{array}$$ (3)

The information exchange matrix [21] is defined as K≜L+C, where *L* is the Laplacian matrix and *C* is defined in Eq (3).

**Lemma 1**. [22] The Laplacian matrix of a component in graph G(ν,ε) is a symmetric matrix with real eigenvalues that satisfy

$$\begin{array}{c} 0=\lambda_{1}\leq \lambda_{2}\leq \lambda_{3}\ldots \lambda_{\zeta }\leq \Delta \end{array}$$

where Δ = 2 × (max_1≤*i*≤*ζ*_ *d*_*i*_).

**Lemma 2**. [18] If at least one agent in each component of graph G has access to the desired signals, then the information exchange matrix *K* = *L* + *C* is symmetric and positive definite.

**Definition 1**. The robust tracking problem is said to be settled if for each *ω* > 0, there is *T* = *T*(*ω*) > 0 and a local distributed control law *τ*_*i*_, *i* ∈ {1, …, *n*}, such that

$$\begin{array}{c} \begin{array}{c} ∥q_{i}\left(t\right)-q_{d}{\left(t\right)∥}_{2}\leq \omega \\ ∥\dot{q_{i}}\left(t\right)-\dot{q_{d}}{\left(t\right)∥}_{2}\leq \omega \\ \forall t\geq t_{0}+T\left(\omega \right) \end{array} \end{array}$$ (4)

in the presence of frictional force and external disturbance.

To facilitate the subsequent analysis, we define

$$\begin{array}{c} x={\left[x_{1}^{T}\;x_{2}^{T}\right]}^{T} \end{array}$$ (5)

$$\begin{array}{c} x_{1}={\left[{\left(q_{1}-q_{d}\right)}^{T}\;,\ldots ,\;{\left(q_{n}-q_{d}\right)}^{T}\right]}^{T} \end{array}$$ (6)

$$\begin{array}{c} x_{2}={\left[{\left({\dot{q}}_{1}-{\dot{q}}_{d}\right)}^{T}\;,\ldots ,\;{\left({\dot{q}}_{n}-{\dot{q}}_{d}\right)}^{T}\right]}^{T} \end{array}$$ (7)

$$\begin{array}{c} e_{i}=\sum_{j\in N_{i}}a_{ij}\left(q_{i}-q_{j}\right)+b_{i}\left(q_{i}-q_{d}\right) \end{array}$$ (8)

$$\begin{array}{c} {\dot{e}}_{i}=\sum_{j\in N_{i}}a_{ij}\left({\dot{q}}_{i}-{\dot{q}}_{j}\right)+b_{i}\left({\dot{q}}_{i}-{\dot{q}}_{d}\right). \end{array}$$ (9)

Before proceeding, we now introduce an important lemma, which will be used in the system stability analysis.

**Lemma 3**. [23] Let *V*(*t*) ≥ 0 be a continuously differentiable function such that $\dot{V}\left(t\right)\leq -\gamma V\left(t\right)+\kappa$, where *γ* and *κ* are positive constants. Then the following inequality is satisfied

$$\begin{array}{c} V\left(t\right)\leq V\left(0\right){\text{e}}^{-\gamma t}+\frac{\kappa }{\gamma }\left(1-{\text{e}}^{-\gamma t}\right). \end{array}$$

## Robust tracking control under fixed topology

### Distributed scheme design

In this subsection, a distributed scheme based on computed-torque control will be given. Computed-torque control is an important approach to decouple complex robotic dynamics, which is shown as follows

$$\begin{array}{c} \tau_{i}=M_{i}\left(q_{i}\right)v_{i}+C_{i}\left(q_{i},{\dot{q}}_{i}\right){\dot{q}}_{i}+G_{i}\left(q_{i}\right) \end{array}$$ (10)

$$\begin{array}{c} v_{i}=b_{i}{\ddot{q}}_{d}-e_{i}-\alpha {\dot{e}}_{i}-\eta_{i} \end{array}$$ (11)

where *α* is a positive constant and *η*_*i*_ is the robust control term. In this paper, *η*_*i*_ is used to estimate the uncertain terms based on information from neighboring agents. Combining Eqs (1), (10) and (11), we get

$$\begin{array}{c} \dot{x}=Fx+H\left(\eta +\gamma \right) \end{array}$$ (12)

where

$$\begin{array}{c} \begin{array}{ccc} F & = & \left[\begin{array}{cc} 0 & I_{n}\\ -K & -\alpha K \end{array}\right]⊗I_{m}\\ H & = & \left[\begin{array}{c} 0\\ -I_{n} \end{array}\right]⊗I_{m}\\ \eta & = & {\left[\eta_{1}^{T}\;,\ldots ,\;\eta_{n}^{T}\right]}^{T}\\ \gamma & = & {\left[\gamma_{1}^{T}\;,\ldots ,\;\gamma_{n}^{T}\right]}^{T}\\ \gamma_{i} & = & \left(b_{i}-1\right){\ddot{q}}_{d}-M_{i}^{-1}\left(q_{i}\right)\left(f_{i}\left({\dot{q}}_{i}\right)+u_{i}\left(t\right)\right). \end{array} \end{array}$$ (13)

For further analysis, we present the following assumptions.

**Assumption 2**. The first order and second order derivatives of the desired trajectory are all bounded (i.e., ${\dot{q}}_{d},{\ddot{q}}_{d}\in L_{\infty }$).

**Assumption 3**. The state velocity ${\dot{q}}_{i}$ is bounded (i.e., ${\dot{q}}_{i}\in L_{\infty }$), and the frictional vector $f_{i}\left({\dot{q}}_{i}\right)$ and its first order and second order derivatives with respect to ${\dot{q}}_{i}$ are bounded (i.e., $f_{i}\left({\dot{q}}_{i}\right),\frac{\partial f_{i}\left({\dot{q}}_{i}\right)}{\partial {\dot{q}}_{i}},\frac{\partial^{2}f_{i}\left({\dot{q}}_{i}\right)}{\partial^{2}{\dot{q}}_{i}}\in L_{\infty }$).

Under Assumption 2 and 3, it is easy to see

$$\begin{array}{ccc} ∥\gamma_{i}{∥}_{\infty } & = & ∥\left(b_{i}-1\right){\ddot{q}}_{d}-M_{i}^{-1}\left(q_{i}\right)\left(f_{i}\left({\dot{q}}_{i}\right)+u_{i}\left(t\right)\right){∥}_{\infty }\\ & \leq & |b_{i}-1|\cdot {∥{\ddot{q}}_{i}∥}_{\infty }+\frac{\left(∥f_{i}\left({\dot{q}}_{i}\right){∥}_{\infty }+∥u_{i}\left(t\right){∥}_{\infty }\right)}{\lambda_{m}}≜\rho_{i}. \end{array}$$ (14)

To facilitate the analysis in the following subsection, we define $\rho ={\left[\rho_{1}^{T},\ldots ,\rho_{n}^{T}\right]}^{T}$ and *χ* = ‖*ρ*‖_2_.

Based on the previous preparations, we present the design of *η*_*i*_ as follows

$$\begin{array}{c} \eta_{i}=\frac{\chi^{2}}{\epsilon }\left(\beta e_{i}+{\dot{e}}_{i}\right) \end{array}$$ (15)

where *ϵ* and *β* are positive parameters which have effects on the convergence precision. The definition of *η*_*i*_ can be utilized to eliminate the effect of frictional vector and external disturbance.

In this section, we consider the fixed topology case where at least one agent in each component has access to the desired signals. By Lemma 2, we know that *K* = *L* + *C* is positive definite, and therefore we can define the smallest and largest eigenvalues of matrix *K* as *λ*_min_(*K*) > 0 and *λ*_max_(*K*) > 0. Before showing our main results, we present the following lemmas, which will be used in the prove of the theorems.

**Lemma 4**. Let $P=[\begin{array}{cc} K & \beta I\\ \beta I & I \end{array}]$ and $Q=[\begin{array}{cc} 2\beta K & K\\ K & 2(\alpha K-\beta I) \end{array}]$, where *α* and *β* are positive parameters. If *α* and *β* satisfy

$$\begin{array}{c} \alpha \beta =1 \end{array}$$ (16)

$$\begin{array}{c} 0<\beta <\frac{\sqrt{3}}{2}\sqrt{\lambda_{\min}\left(K\right)} \end{array}$$ (17)

then *P* and *Q* are both positive definite.

*Proof:* Let *λ*_*i*_, *i* = {1, …, *n*}, denote the *n* eigenvalues of *K*. We know the eigenvalue *s* of *P* satisfies

$$\begin{array}{c} s^{2}-\left(\lambda_{i}+1\right)s+\lambda_{i}-\beta^{2}=0. \end{array}$$ (18)

Obviously, *λ*_*i*_ + 1 > 0 and *λ*_*i*_ − *β*^2^ > 0, which imply the roots of Eq (18) are both positive. Thus *P* is positive definite. Similarly, the eigenvalue *ω* of *Q* satisfies

$$\begin{array}{c} \omega^{2}-2\left(\beta \lambda_{i}+\alpha \lambda_{i}-\beta \right)\omega +4\alpha \beta \lambda_{i}^{2}-4\beta^{2}\lambda_{i}-\lambda_{i}^{2}=0. \end{array}$$ (19)

We have

$$\begin{array}{c} 2\left(\beta \gamma_{i}+\alpha \gamma_{i}-\beta \right)=\frac{2}{\beta }\left(\beta^{2}\gamma_{i}+\gamma_{i}-\beta^{2}\right)>\frac{2}{\beta }\left(\gamma_{i}-\beta^{2}\right)>0 \end{array}$$ (20)

$$\begin{array}{c} 4\alpha \beta \gamma_{i}^{2}-4\beta^{2}\gamma_{i}-\gamma_{i}^{2}=3\gamma_{i}^{2}-4\beta^{2}\gamma_{i}=3\gamma_{i}\left(\gamma_{i}-\frac{4}{3}\beta^{2}\right)>0. \end{array}$$ (21)

Thus the roots of Eq (19) are positive, i.e., *Q* is positive definite.

*Remark 1:* The Assumptions used in this paper are idiomatically in the study of physical systems described by Euler-Lagrange equation. The elements of matrix *M*_*i*_(*q*_*i*_) are rotary inertias of the joints and the readers can refer to [19, 20] for the precise algebraic expressions. An important property is that *M*_*i*_(*q*_*i*_) is positive definite and bounded. Assumptions 2 and 3 mention that the physical parameters and desired trajectory are all bounded, which are naturally in the dynamic behaviour of physical systems [15]. Assumptions 2 and 3 are basically referring to the Lipschits condition. If $∥f_{i}\left({\dot{q}}_{i}+\Delta \right)-f_{i}\left({\dot{q}}_{i}\right)∥\leq L∥\Delta ∥$, then we can see that $\frac{\partial f_{i}\left({\dot{q}}_{i}\right)}{\partial {\dot{q}}_{i}}\in L_{\infty }$.

### Convergence analysis

**Theorem 1**. Consider *n* Euler-Lagrange systems described as Eq (1). Let Assumptions 1, 2 and 3 be fulfilled. Suppose the interaction topology is fixed and at least one agent in each component has access to the desired signals. Then, under the control strategy Eqs (10), (11) and (15), for any sufficiently small constant *ϵ* > 0 and $\bar{\epsilon }=\sqrt{\frac{1}{\lambda_{\min}\left(P\right)}\left(1+\frac{1}{2\lambda_{\min}\left(K\right)\psi }\right)\epsilon }$, there is

$$\begin{array}{c} T\left(\epsilon \right)=\frac{\lambda_{\max}\left(P\right)}{\lambda_{\min}\left(Q\right)}\ln\frac{V(t_{0})}{\epsilon } \end{array}$$

such that

$$\begin{array}{c} {∥x\left(t\right)∥}_{2}\leq \bar{\epsilon },\;\forall t\geq t_{0}+T\left(\epsilon \right) \end{array}$$ (22)

where *V*(*t*_0_) = *x*^*T*^(*t*_0_)(*P* ⊗ *I*_*m*_)*x*(*t*_0_) and the control parameters satisfy

$$\begin{array}{c} \alpha \beta =1 \end{array}$$ (23)

$$\begin{array}{c} 0<\beta <\frac{\sqrt{3}}{2}\sqrt{\lambda_{\min}\left(K\right)}. \end{array}$$ (24)

*Proof:* Take the Lyapunov function $V\left(t\right)=x^{T}\left(t\right)\bar{P}x\left(t\right)$, where

$$\begin{array}{c} \bar{P}=P⊗I_{m}. \end{array}$$ (25)

The derivative of *V*(*t*) along the solutions of the closed-loop system is

$$\begin{array}{ccc} \dot{V} & = & x^{T}\left(F^{T}\bar{P}+\bar{P}F\right)x+2x^{T}\bar{P}H\left(\eta +\gamma \right)\\ & = & -x^{T}\left(Q⊗I_{m}\right)x+2x^{T}\bar{P}H\left(\eta +\gamma \right)\\ & \leq & -x^{T}\left(Q⊗I_{m}\right)x+2x^{T}\bar{P}H\eta +2\chi {∥H^{T}\bar{P}x∥}_{2}. \end{array}$$ (26)

From Eq (15), we have

$$\begin{array}{ccc} x^{T}\bar{P}H\eta & = & \frac{\chi^{2}}{\epsilon }x^{T}\bar{P}H\left(\beta e+\dot{e}\right)\\ & = & \frac{\chi^{2}}{\epsilon }x^{T}\bar{P}H\left(\left(\beta K⊗I_{m}\right)x_{1}+\left(K⊗I_{m}\right)x_{2}\right)\\ & = & \frac{\chi^{2}}{\epsilon }x^{T}\bar{P}H\left(\left[\begin{array}{cc} \beta K & K \end{array}\right]⊗I_{m}\right)x\\ & = & -\frac{\chi^{2}}{\epsilon }x^{T}\bar{P}H\left(\left(K\left[\begin{array}{cc} 0 & -I_{n} \end{array}\right]P\right)⊗I_{m}\right)x\\ & = & -\frac{\chi^{2}}{\epsilon }x^{T}\bar{P}H\left(K⊗I_{m}\right)H^{T}\bar{P}x\\ & \leq & -\frac{\lambda_{\min}\left(K\right)\chi^{2}}{\epsilon }{∥H^{T}\bar{P}x∥}_{2}^{2}. \end{array}$$ (27)

Substituting Eq (27) into Eq (26) yields

$$\begin{array}{ccc} \dot{V} & \leq & -x^{T}\left(Q⊗I_{m}\right)x-\frac{2\lambda_{\min}\left(K\right)\chi^{2}}{\epsilon }∥H^{T}\bar{P}{x∥}_{2}^{2}+2\chi {∥H^{T}\bar{P}x∥}_{2}\\ & \leq & -x^{T}\left(Q⊗I_{m}\right)x-{\left(\frac{\sqrt{2\lambda_{\min}\left(K\right)}\chi }{\sqrt{\epsilon }}∥H^{T}\bar{P}x{∥}_{2}-\frac{\sqrt{\epsilon }}{\sqrt{2\lambda_{\min}\left(K\right)}}\right)}^{2}+\frac{\epsilon }{2\lambda_{\min}\left(K\right)}\\ & \leq & -x^{T}\left(Q⊗I_{m}\right)x+\frac{\epsilon }{2\lambda_{\min}\left(K\right)}\\ & \leq & -\psi V+\frac{\epsilon }{2\lambda_{\min}\left(K\right)} \end{array}$$ (28)

where $\psi =\frac{\lambda_{\min}\left(Q\right)}{\lambda_{\max}\left(P\right)}$. By Lemma 3, we have

$$\begin{array}{ccc} V(t) & \leq & {\text{e}}^{-\psi (t-t_{0})}V\left(t_{0}\right)+\frac{\epsilon }{2\lambda_{\min}\left(K\right)\psi }\left(1-{\text{e}}^{-\psi (t-t_{0})}\right)\\ & \leq & {\text{e}}^{-\psi (t-t_{0})}V\left(t_{0}\right)+\frac{\epsilon }{2\lambda_{\min}\left(K\right)\psi }. \end{array}$$ (29)

Therefore, for any $t\geq t_{0}+\frac{1}{\psi }\ln\frac{V(t_{0})}{\epsilon }$, we can get that

$$\begin{array}{c} V\left(t\right)\leq \left(1+\frac{1}{2\lambda_{\min}\left(K\right)\psi }\right)\epsilon \end{array}$$ (30)

i.e.,

$$\begin{array}{c} {∥x∥}_{2}\leq \sqrt{\frac{1}{\lambda_{\min}\left(P\right)}\left(1+\frac{1}{2\lambda_{\min}\left(K\right)\psi }\right)\epsilon }=\bar{\epsilon }. \end{array}$$ (31)

It means for any *ϵ* > 0, there is $T\left(\epsilon \right)=\frac{1}{\psi }\ln\frac{V(t_{0})}{\epsilon }$ such that

$$\begin{array}{c} {∥x\left(t\right)∥}_{2}\leq \bar{\epsilon },\;\forall t\geq t_{0}+T\left(\epsilon \right) \end{array}$$ (32)

which completes the proof.

**Fig 1:**
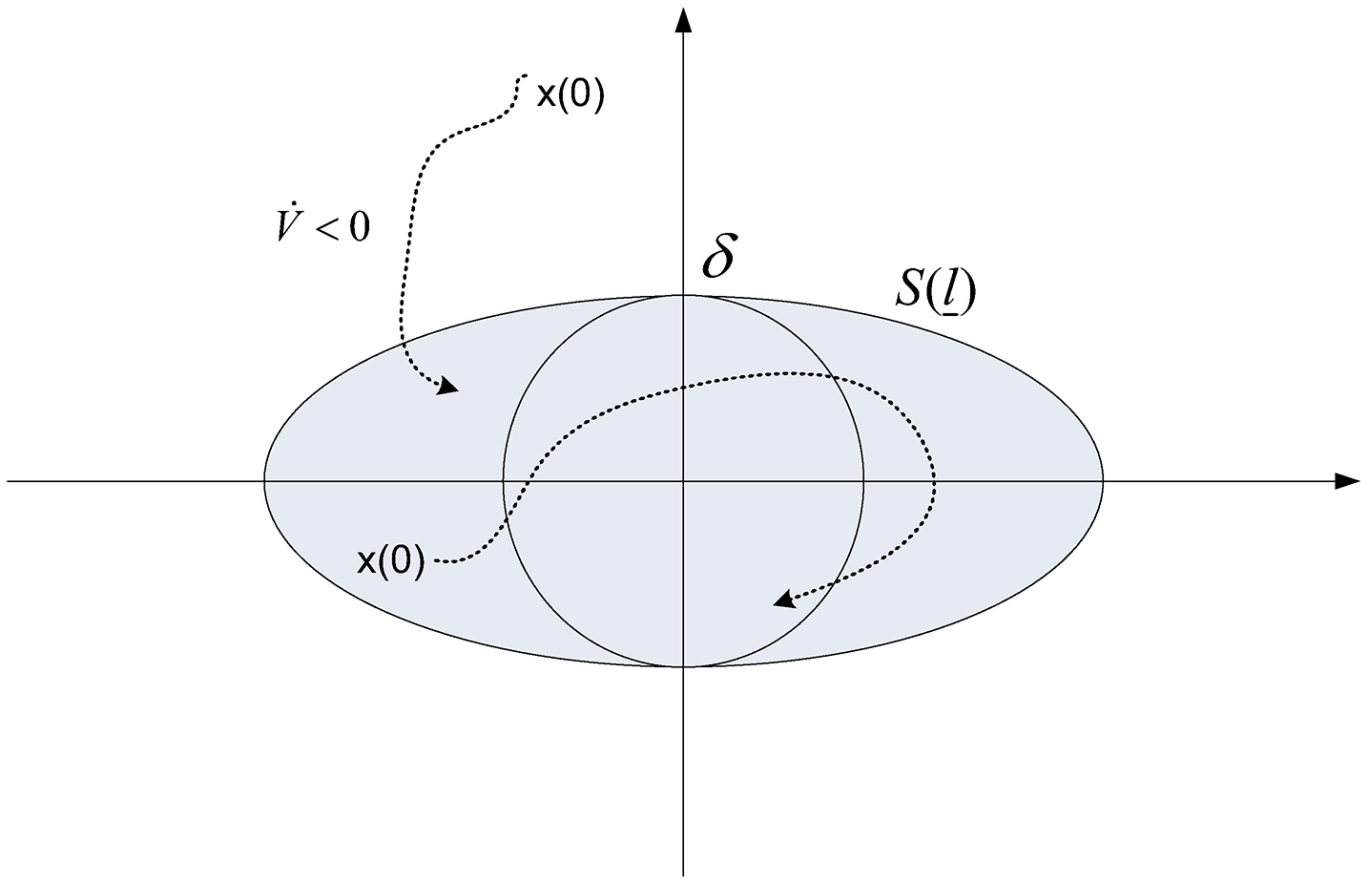
The illustration for trajectory x(t).

*Remark 2:* In contrast to the proof of Theorem 1, where algebraic approaches are conducted, we now give a geometric proof based on Fig 1.

The Eq (28) shows that $\dot{V}\leq -x^{T}\left(Q⊗I_{m}\right)x+\frac{\epsilon }{2\lambda_{\min}\left(K\right)}$ which implies $\dot{V}<0$ for ${∥x∥}_{2}>\frac{\sqrt{\epsilon }}{\sqrt{2\lambda_{\min}\left(Q\right)\lambda_{\min}\left(K\right)}}≜\delta$.

Let’s define

$$\begin{array}{ccc} B_{\delta } & = & \left\{x|∥x{∥}_{2}\leq \delta \right\}\\ S(l) & = & \left\{x|x^{T}\bar{P}x\leq l\right\}\\ \underline{l} & = & \min\{l|S\left(l\right)⊃B_{\delta }\} \end{array}$$ (33)

i.e., $S(\underline{l})$ is the minimum ellipsoid containing *B*_*δ*_. Next, we will show that for any initial value *x*(*t*_0_), the trajectory *x*(*t*) converges to $S(\underline{l})$ in finite time.

Obviously, $x\left(t\right)\in S\left(\underline{l}\right),\;\forall t\geq t_{0}$ for any $x\left(t_{0}\right)\in S\left(\underline{l}\right)$. Thus in the following, we just need to consider the case when $x\left(t_{0}\right)\notin S\left(\underline{l}\right)$. Let $k_{0}=x^{T}\left(t_{0}\right)\bar{P}x\left(t_{0}\right)$ and $c_{0}=\min\left\{x^{T}\left(Q⊗I_{m}\right)x-\frac{\epsilon }{2\lambda_{\min}\left(K\right)}|x\in S\left(k_{0}\right)-S\left(\underline{l}\right)\right\}$. By Eq (28), we have

$$\begin{array}{c} \int_{t_{0}}^{t}\dot{V}dt\leq -c_{0}\left(t-t_{0}\right). \end{array}$$ (34)

Thus, the time point *t*_1_ when *V*(*t*) reaches the boundary of the ellipsoid $S(\underline{l})$ satisfies

$$\begin{array}{c} t_{1}\leq t_{0}+\frac{k_{0}-\underline{l}}{c_{0}}. \end{array}$$ (35)

Then *V*(*t*) will be limited in the bounded ellipsoid $S(\underline{l})$ for any *t* ≥ *t*_1_. Thus, we have

$$\begin{array}{c} \lambda_{\min}\left(P\right){∥x\left(t\right)∥}_{2}^{2}\leq V\left(t\right)\leq \underline{l},\;\forall t\geq t_{1} \end{array}$$ (36)

i.e., ${∥x\left(t\right)∥}_{2}\leq \frac{\underline{l}}{\lambda_{\min}\left(P\right)},\forall t\geq t_{1}$. It means that the norm of the trajectory error vector can be reduced to any prescribed positive value.

## Robust tracking control under switching topologies

### Distributed scheme design

In this section, we extend the results in the above section to the switching topology case, where the switching signal is chosen as σ(t):[0,∞)→M. Consider an infinite sequence of nonempty, bounded, and contiguous time-intervals [*t*_*r*_, *t*_*r*+1_), *r* = 0, 1, … with *t*_0_ = 0, *t*_*r*+1_ ≤ *t*_*r*_ + *T* for a constant *T* > 0. In each interval [*t*_*r*_, *t*_*r*+1_), there is a sequence of subintervals

$$\begin{array}{c} \left[t_{r}^{0},t_{r}^{1}\right),\left[t_{r}^{1},t_{r}^{2}\right),\ldots ,\left[t_{r}^{m_{r}-1},t_{r}^{m_{r}}\right);\;t_{r}=t_{r}^{0},t_{r+1}=t_{r}^{m_{r}} \end{array}$$

satisfying $t_{r}^{j+1}-t_{r}^{j}\geq \tau ,0\leq j\leq m_{r}-1$, for some integer *m*_*r*_ ≥ 0 and a given constant *τ* > 0, such that the interaction graph $G_{\sigma (t)}$ switches at $t_{r}^{j}$ and does not change during each subinterval $\left[t_{r}^{j},t_{r}^{j+1}\right)$.

Suppose the interaction graph $G_{\sigma }$ in subinterval $\left[t_{r}^{j},t_{r}^{j+1}\right)$ has *l*_*σ*_ ≥ 1 connected components with the corresponding node numbers denoted by $\theta_{\sigma }^{1},\ldots ,\theta_{\sigma }^{l_{\sigma }}$. For simplicity, we suppose that the first *h*(1 ≤ *h* ≤ *l*_*σ*_) components have accesses to the desired signals. Then, by Lemmas 1 and 2, we know that matrix *K*_*σ*_ = *L*_*σ*_ + *B*_*σ*_ is semi-positive definite and there is matrix $S_{\sigma }\in R^{n\times n},S_{\sigma }^{T}S_{\sigma }=I$, such that

$$\begin{array}{c} S_{\sigma }^{T}K_{\sigma }S_{\sigma }=\text{diag}\left\{K_{\sigma }^{1},K_{\sigma }^{2},\ldots ,K_{\sigma }^{l_{\sigma }}\right\}≜\Lambda_{\sigma } \end{array}$$ (37)

where

$$\begin{array}{c} \left\{ \begin{array}{ccc} K_{\sigma }^{i} & = & \text{diag}\{\lambda_{\sigma ,1}^{i},\lambda_{\sigma ,2}^{i},\ldots ,\lambda_{\sigma ,\theta_{\sigma }^{i}}^{i}\},\;1\leq i\leq h;\\ K_{\sigma }^{i} & = & \text{diag}\left\{0,\lambda_{\sigma ,2}^{i},\ldots ,\lambda_{\sigma ,\theta_{\sigma }^{i}}^{i}\right\},\;h<i\leq l_{\sigma }. \end{array}\right. \end{array}$$ (38)

We define

$$\begin{array}{c} \left\{ \begin{array}{ccc} {\hat{K}}_{\sigma }^{i} & = & \text{diag}\{\lambda_{\sigma ,1}^{i},\lambda_{\sigma ,2}^{i},\ldots ,\lambda_{\sigma ,\theta_{\sigma }^{i}}^{i}\},1\leq i\leq h;\\ {\hat{K}}_{\sigma }^{i} & = & \text{diag}\left\{\lambda_{\sigma ,2}^{i},\lambda_{\sigma ,2}^{i},\ldots ,\lambda_{\sigma ,\theta_{\sigma }^{i}}^{i}\right\},h<i\leq l_{\sigma };\\ {\hat{\Lambda }}_{\sigma } & = & \text{diag}\{{\hat{K}}_{\sigma }^{1},{\hat{K}}_{\sigma }^{2},\ldots ,{\hat{K}}_{\sigma }^{l_{\sigma }}\}. \end{array}\right. \end{array}$$ (39)

It is easy to see that

$$\begin{array}{ccc} K_{\sigma } & = & S_{\sigma }\Lambda_{\sigma }S_{\sigma }^{T}\\ & = & S_{\sigma }\sqrt{\Lambda_{\sigma }}\sqrt{\hat{\Lambda_{\sigma }}}{\hat{\Lambda }}_{\sigma }^{-1}\sqrt{\hat{\Lambda_{\sigma }}}\sqrt{\Lambda_{\sigma }}S_{\sigma }^{T}\\ & = & S_{\sigma }\sqrt{\Lambda_{\sigma }}\sqrt{\Lambda_{\sigma }}{\hat{\Lambda }}_{\sigma }^{-1}\sqrt{\Lambda_{\sigma }}\sqrt{\Lambda_{\sigma }}S_{\sigma }^{T}\\ & = & \left(S_{\sigma }\Lambda_{\sigma }S_{\sigma }^{T}\right)\left(S_{\sigma }{\hat{\Lambda }}_{\sigma }^{-1}S_{\sigma }^{T}\right)\left(S_{\sigma }\Lambda_{\sigma }S_{\sigma }^{T}\right)\\ & = & K_{\sigma }\Phi_{\sigma }K_{\sigma } \end{array}$$ (40)

where $\Phi_{\sigma }=S_{\sigma }{\hat{\Lambda }}_{\sigma }^{-1}S_{\sigma }^{T}$ is positive definite.

In this case, the distributed robust tracking algorithm for Eq (1) is defined as

$$\begin{array}{c} \tau_{i}=M_{i}\left(q_{i}\right)v_{i}+C_{i}\left(q_{i},{\dot{q}}_{i}\right){\dot{q}}_{i}+G_{i}\left(q_{i}\right) \end{array}$$ (41)

$$\begin{array}{c} v_{i}=b_{i}{\ddot{q}}_{d}+\mu b_{i}{\dot{q}}_{d}-\mu {\dot{q}}_{i}-de_{i}-d{\dot{e}}_{i}-{\bar{\eta }}_{i} \end{array}$$ (42)

where *μ* > 0 and *d* > 0 are positive constants; ${\bar{\eta }}_{i}$ is the robust input term used to eliminate the effect of uncertain terms, which will be shown in the following part. Combining Eqs (1), (41) and (42), we get

$$\begin{array}{c} \dot{x}=Z_{\sigma (t)}x+E\left(\bar{\eta }+\bar{\gamma }\right) \end{array}$$ (43)

where

$$\begin{array}{ccc} Z_{\sigma (t)} & = & \left[\begin{array}{cc} 0 & I_{n}\\ -dK_{\sigma (t)} & -\mu I-dK_{\sigma (t)} \end{array}\right]⊗I_{m}\\ E & = & \left[\begin{array}{c} 0\\ -I_{n} \end{array}\right]⊗I_{m}\\ \bar{\eta } & = & {\left[{\bar{\eta }}_{1}^{T}\;,\ldots ,\;{\bar{\eta }}_{n}^{T}\right]}^{T}\\ \bar{\gamma } & = & {\left[{\bar{\gamma }}_{1}^{T}\;,\ldots ,\;{\bar{\gamma }}_{n}^{T}\right]}^{T}\\ {\bar{\gamma }}_{i} & = & \left(b_{i}-1\right){\ddot{q}}_{d}+\mu \left(b_{i}-1\right){\dot{q}}_{d}-M_{i}^{-1}\left(q_{i}\right)\left(f_{i}\left({\dot{q}}_{i}\right)+u_{i}\left(t\right)\right). \end{array}$$ (44)

Similar to Eq (14), we know ${\bar{\gamma }}_{i}$ is upper bounded and satisfies the following inequality

$$\begin{array}{ccc} ∥{\bar{\gamma }}_{i}{∥}_{\infty } & = & ∥\left(b_{i}-1\right){\ddot{q}}_{d}-\mu \left(b_{i}-1\right){\dot{q}}_{d}-M_{i}^{-1}\left(q_{i}\right)\left(f_{i}\left({\dot{q}}_{i}\right)+u_{i}\left(t\right)\right){∥}_{\infty }\\ & \leq & |b_{1}-1|(∥{\ddot{q}}_{d}{∥}_{\infty }+\mu ∥{\dot{q}}_{d}{∥}_{\infty })+\frac{1}{\lambda_{m}}(∥f_{i}\left({\dot{q}}_{i}\right){∥}_{\infty }+∥u_{i}\left(t\right){∥}_{\infty })≜{\bar{\rho }}_{i}. \end{array}$$ (45)

Thus $\bar{\gamma }={\left[{\bar{\gamma }}_{1}^{T}\;,\ldots ,\;{\bar{\gamma }}_{n}^{T}\right]}^{T}$ is upper bounded (i.e., $\bar{\gamma }\in L_{\infty }$). A necessary requirement in the investigation of multi-agent systems under switching topology is that there is a bounded piecewise continuous vector *ϕ*_*σ*(*t*)_ satisfying

$$\begin{array}{c} \bar{\gamma }=\left(K_{\sigma (t)}⊗I_{m}\right)\phi_{\sigma (t)} \end{array}$$ (46)

where the upper bound of *ϕ*_*σ*(*t*)_ is denoted by *φ* (i.e., ‖*ϕ*_*σ*(*t*)_‖_∞_ ≤ *φ*).

Based on the preparations, the robust input term is defined as

$$\begin{array}{c} {\bar{\eta }}_{i}=\frac{\varphi^{2}}{2\epsilon }\left(e_{i}+{\dot{e}}_{i}\right). \end{array}$$ (47)

It is emphasized that *φ* is the upper bound of the uncertain terms and *ϵ* is a design parameter which has an effect on the consensus precision. From Eq (47) we can see that there is significant positive correlation between the control energy and *φ*, and there is a significant negative correlation between the control energy and *ϵ*.

The following lemma will be used in the robust convergence analysis.

**Lemma 5**. Let $D=[\begin{array}{cc} \mu I & I\\ I & I \end{array}]$ and $Q_{\sigma (t)}=[\begin{array}{cc} 2dK_{\sigma (t)} & 2dK_{\sigma (t)}\\ 2dK_{\sigma (t)} & 2(\mu I+dK_{\sigma (t)}-I) \end{array}]$, where *μ* and *d* are positive parameters. If *μ* > 1, then *D* is positive definite and *Q*_*σ*(*t*)_ is positive semi-definite for ∀*t* ∈ [0, ∞). Furthermore, *Q*_*σ*(*t*)_ > 0 if and only if *K*_*σ*_(*t*) > 0.

*Proof:* The fact that *D* > 0 is obvious and the proof is omitted here. By Eq (37), we know *K*_*σ*_ can be transformed into a diagonal matrix as $K_{\sigma }=S_{\sigma }\Lambda_{\sigma }S_{\sigma }^{T}$. For simplicity, we redefine Λ_*σ*_ as $\Lambda_{\sigma }=\text{diag}\left\{\lambda_{\sigma }^{1},\ldots ,\lambda_{\sigma }^{n}\right\}$, where $\lambda_{\sigma }^{i}\geq 0$ is the *i*th eigenvalue of *K*_*σ*_. Then *Q*_*σ*_ can be written as

$$\begin{array}{ccc} Q_{\sigma } & = & \left[\begin{array}{cc} S_{\sigma } & 0_{n\times n}\\ 0_{n\times n} & S_{\sigma } \end{array}\right]\left[\begin{array}{cc} 2d\Lambda_{\sigma } & 2d\Lambda_{\sigma }\\ 2d\Lambda_{\sigma } & 2(\left(\mu -1\right)I+d\Lambda_{\sigma }) \end{array}\right]\left[\begin{array}{cc} S_{\sigma }^{T} & 0_{n\times n}\\ 0_{n\times n} & S_{\sigma }^{T} \end{array}\right]. \end{array}$$ (48)

For any eigenvalue *ω*_*σ*_ of *Q*_*σ*_, we have that

$$\begin{array}{c} \left(\omega_{\sigma }-2d\lambda_{\sigma }^{i}\right)\left(\omega_{\sigma }-2\left(\mu -1+d\lambda_{\sigma }^{i}\right)\right)-{\left(2d\lambda_{\sigma }^{i}\right)}^{2}=0 \end{array}$$ (49)

i.e.,

$$\begin{array}{c} \omega_{\sigma }^{2}-2\left(\mu -1+2d\lambda_{\sigma }^{i}\right)\omega_{\sigma }+4\left(\mu -1\right)d\lambda_{\sigma }^{i}=0. \end{array}$$ (50)

For any *μ* > 1 and *d* > 0, we know $2(\mu -1+2d\lambda_{\sigma }^{i})>0$ and $4\left(\mu -1\right)d\lambda_{\sigma }^{i}\geq 0$. It follows that *ω*_*σ*_ ≥ 0, i.e., *Q*_*σ*_ is positive semi-definite. Furthermore, *ω*_*σ*_ > 0 if and only if $4\left(\mu -1\right)d\lambda_{\sigma }^{i}>0,i=\left\{1,2,\ldots ,n\right\}$, i.e., *K*_*σ*_ > 0.

### Convergence analysis

In this subsection, we will prove that the closed-loop system could maintain a satisfactory performance with switching topologies. Before giving the main result, we will present some preliminary definitions. Note that σ(t):[0,∞)→M is the finite switching signal and does not change during the time intervals no less than *τ*. We define $\xi =\min_{\sigma (t)\in M}\lambda_{\min}\left(\Phi_{\sigma (t)}\right)$, $\varrho =\min_{\sigma (t)\in M}\lambda_{\min}\left(Q_{\sigma (t)}\right)$, and $\nu =\frac{\varrho }{\lambda_{\max}\left(D\right)}$, where *D* is defined in Lemma 5.

**Theorem 2**. Consider *n* Euler-Lagrange systems described as Eq (1) with switching interaction topologies. Let Assumptions 1, 2 and 3 be fulfilled. Suppose that during each time interval [*t*_*r*_, *t*_*r*+1_), *t*_*r*+1_ ≤ *t*_*r*_ + *T*, there is one subinterval $\left[t_{r}^{j},t_{r}^{j+1}\right)$ such that all the components have accesses to the desired signals. Then, under the control strategy Eqs (41), (42) and (47), for any sufficiently small constant *ϵ* > 0 and $\bar{\epsilon }=\sqrt{\frac{1}{\lambda_{\min}\left(D\right)}\left(1+\frac{T}{\xi }+\frac{1+\nu \tau }{(1-{\text{e}}^{-\nu \tau })\nu \xi }\right)\epsilon }$, there is

$$\begin{array}{c} \chi \left(\epsilon \right)=\left(1+\frac{1}{\nu \tau }\ln\frac{V(0)}{\epsilon }\right)T \end{array}$$

such that

$$\begin{array}{c} {∥x\left(t\right)∥}_{2}\leq \bar{\epsilon },\;\forall t\geq \chi \left(\epsilon \right) \end{array}$$ (51)

where *V*(0) = *x*^*T*^(0)(*D* ⊗ *I*_*m*_)*x*(0) and the control parameters satisfy

$$\begin{array}{c} \mu >1 \end{array}$$ (52)

$$\begin{array}{c} d>0. \end{array}$$ (53)

*Proof:* Take the Lyapunov function $V\left(t\right)=x^{T}\left(t\right)\bar{D}x\left(t\right)$, where

$$\begin{array}{c} \bar{D}=D⊗I_{m}. \end{array}$$ (54)

We can see that *V*(*t*) is piecewise differentiable and the derivative of *V*(*t*) along the solutions of the closed-loop system during $\left[t_{r}^{j},t_{r}^{j+1}\right)$ is

$$\begin{array}{ccc} \dot{V}\left(t\right) & = & x^{T}\left(Z_{\sigma (t)}^{T}\bar{D}+\bar{D}Z_{\sigma (t)}\right)x+2x^{T}\bar{D}E\left(\bar{\eta }+\bar{\gamma }\right)\\ & = & -x^{T}\left(Q_{\sigma (t)}⊗I_{m}\right)x+2x^{T}\bar{D}E\bar{\eta }+2x^{T}\bar{D}EK_{\sigma (t)}\varphi_{\sigma (t)}\\ & \leq & -x^{T}\left(Q_{\sigma (t)}⊗I_{m}\right)x+2x^{T}\bar{D}E\bar{\eta }+2\varphi {∥\left(K_{\sigma (t)}⊗I_{m}\right)E^{T}\bar{D}x∥}_{2}. \end{array}$$ (55)

From Eqs (40) and (47), we can get that

$$\begin{array}{ccc} x^{T}\bar{D}E\bar{\eta } & = & \frac{\varphi^{2}}{2\epsilon }x^{T}\bar{D}E\left(e+\dot{e}\right)\\ & = & -\frac{\varphi^{2}}{2\epsilon }x^{T}\bar{D}E\left(K_{\sigma (t)}⊗I_{m}\right)E^{T}\bar{D}x\\ & = & -\frac{\varphi^{2}}{2\epsilon }x^{T}\bar{D}E\left(K_{\sigma (t)}⊗I_{m}\right)\left(\Phi_{\sigma (t)}⊗I_{m}\right)\left(K_{\sigma (t)}⊗I_{m}\right)E^{T}\bar{D}x\\ & \leq & -\frac{\xi \varphi^{2}}{2\epsilon }{∥\left(K_{\sigma (t)}⊗I_{m}\right)E^{T}\bar{D}x∥}_{2}^{2}. \end{array}$$ (56)

Thus, we have

$$\begin{array}{ccc} \dot{V}\left(t\right) & \leq & -x^{T}\left(Q_{\sigma (t)}⊗I_{m}\right)x-(\frac{\sqrt{\xi }\varphi }{\sqrt{\epsilon }}∥{\left(K_{\sigma (t)}⊗I_{m}\right)E^{T}\bar{D}x{∥}_{2}-\frac{\sqrt{\epsilon }}{\sqrt{\xi }})}^{2}+\frac{\epsilon }{\xi }\\ & \leq & -x^{T}\left(Q_{\sigma (t)}⊗I_{m}\right)x+\frac{\epsilon }{\xi }\\ & \leq & -\nu V\left(t\right)+\frac{\epsilon }{\xi }. \end{array}$$ (57)

According to Lemma 3, we obtain

$$\begin{array}{ccc} V(t_{r}^{j+1}) & \leq & {\text{e}}^{-\nu (t_{r}^{j+1}-t_{r}^{j})}V\left(t_{r}^{j}\right)+\frac{\epsilon }{\nu \xi }\left(1-{\text{e}}^{-\nu (t_{r}^{j+1}-t_{r}^{j})}\right)\\ & \leq & {\text{e}}^{-\nu (t_{r}^{j+1}-t_{r}^{j})}V\left(t_{r}^{j}\right)+\frac{\epsilon }{\nu \xi }. \end{array}$$ (58)

For any other subinterval $[t_{r}^{i},t_{r}^{i+1}),i\neq j$, in which not all the components have accesses to the desired signals, we have $\dot{V}\left(t\right)\leq \frac{\epsilon }{\xi }$. It follows that

$$\begin{array}{c} V\left(t_{r}^{i+1}\right)\leq V\left(t_{r}^{i}\right)+\frac{\epsilon }{\xi }\left(t_{r}^{i+1}-t_{r}^{i}\right). \end{array}$$ (59)

Thus, we get

$$\begin{array}{ccc} V(t_{r+1}) & \leq & V\left(t_{r}^{j+1}\right)+\frac{\epsilon }{\xi }\left(t_{r+1}-t_{r}^{j+1}\right)\\ & \leq & {\text{e}}^{-\nu (t_{r}^{j+1}-t_{r}^{j})}V\left(t_{r}^{j}\right)+\frac{\epsilon }{\nu \xi }+\frac{\epsilon }{\xi }\left(t_{r+1}-t_{r}^{j+1}\right)\\ & \leq & {\text{e}}^{-\nu (t_{r}^{j+1}-t_{r}^{j})}\left(V\left(t_{r}\right)+\frac{\epsilon }{\xi }\left(t_{r}^{j}-t_{r}\right)\right)+\frac{\epsilon }{\nu \xi }+\frac{\epsilon }{\xi }\left(t_{r+1}-t_{r}^{j+1}\right)\\ & \leq & {\text{e}}^{-\nu \tau }V\left(t_{r}\right)+\left(\frac{T}{\xi }+\frac{1}{\nu \xi }\right)\epsilon . \end{array}$$ (60)

It follows that

$$\begin{array}{ccc} V(t_{r+1}) & \leq & {\text{e}}^{-(r+1)\nu \tau }V\left(0\right)+\left({\text{e}}^{-r\nu \tau }+{\text{e}}^{-(r-1)\nu \tau }+\ldots +1\right)\left(\frac{T}{\xi }+\frac{1}{\nu \xi }\right)\epsilon \\ & \leq & {\text{e}}^{-(r+1)\nu \tau }V\left(0\right)+\frac{1+\nu T}{(1-{\text{e}}^{-\nu \tau })\nu \xi }\epsilon . \end{array}$$ (61)

Therefore, for any *t*_*r*_ < *t* < *t*_*r*+1_ we have

$$\begin{array}{ccc} V(t) & \leq & V\left(t_{r}\right)+\frac{\epsilon }{\xi }T\\ & \leq & {\text{e}}^{-r\nu \tau }V\left(0\right)+\left(\frac{T}{\xi }+\frac{1+\nu \tau }{(1-{\text{e}}^{-\nu \tau })\nu \xi }\right)\epsilon \\ & \leq & {\text{e}}^{-\nu \tau [\frac{t}{T}]}V\left(0\right)+\left(\frac{T}{\xi }+\frac{1+\nu \tau }{(1-{\text{e}}^{-\nu \tau })\nu \xi }\right)\epsilon \end{array}$$ (62)

where $\left[\frac{t}{T}\right]$ is the integer part of $\frac{t}{T}$ which satisfies $\left[\frac{t}{T}\right]>\frac{t}{T}-1$.

Obviously, for any $t\geq (1+\frac{1}{\nu \tau }\ln\frac{V(0)}{\epsilon })T$, we have

$$\begin{array}{ccc} V(t) & \leq & {\text{e}}^{-\nu \tau [\frac{t}{T}]}V\left(0\right)+\left(\frac{T}{\xi }+\frac{1+\nu \tau }{(1-{\text{e}}^{-\nu \tau })\nu \xi }\right)\epsilon \\ & < & {\text{e}}^{-\nu \tau (\frac{t}{T}-1)}V\left(0\right)+\left(\frac{T}{\xi }+\frac{1+\nu \tau }{(1-{\text{e}}^{-\nu \tau })\nu \xi }\right)\epsilon \\ & \leq & (1+\frac{T}{\xi }+\frac{1+\nu \tau }{(1-{\text{e}}^{-\nu \tau })\nu \xi })\epsilon . \end{array}$$ (63)

It follows that

$$\begin{array}{c} \lambda_{\min}\left(D\right){∥x\left(t\right)∥}_{2}^{2}\leq V\left(t\right)<\left(1+\frac{T}{\xi }+\frac{1+\nu \tau }{(1-{\text{e}}^{-\nu \tau })\nu \xi }\right)\epsilon . \end{array}$$ (64)

By Eq (50), we have

$$\begin{array}{c} {∥x∥}_{2}<\sqrt{\frac{1}{\lambda_{\min}\left(D\right)}\left(1+\frac{T}{\xi }+\frac{1+\nu \tau }{(1-{\text{e}}^{-\nu \tau })\nu \xi }\right)\epsilon }=\bar{\epsilon }. \end{array}$$ (65)

We conclude that for any given $\bar{\epsilon }$, there is $\chi \left(\bar{\epsilon }\right)=\left(1+\frac{1}{\nu \tau }\ln\frac{V(0)}{\epsilon }\right)T$ such that

$$\begin{array}{c} {∥x∥}_{2}\leq \bar{\epsilon },\;\forall t\geq \chi \left(\epsilon \right). \end{array}$$ (66)

This completes the proof.

*Remark 3:* Our distributed protocols are based on computed torque approach which is a useful method to linearize robotic dynamics. Many researchers have made great efforts in the investigation of computed torque control method and considerable control strategies have been proposed. In [24, 25], adaptive control methods were proposed for mechanical manipulators based on computed torque control. In [26], a variable structure controller was considered for computed torque approach. These works made computed torque control widely used in mechanical systems.

*Remark 4:* Compared with existing results, where consensus problems with switching topologies are considered, our contribution of this paper is threefold. First, mechanical systems with nonlinear dynamic models are investigated in this paper. In [10], consensus problem of first-order multi-agent systems was well settled under switching topologies, where matrix theory and algebraic graph theory were used. However, their method can not be extended into consensus problem of nonlinear systems. Different from [10], a Liapunov based approach is provided in this paper. Second, most of the results (see [27] for instance) on this topic require that all the subgraphs are connected, i.e., the switching signal switches between connected subgraphs. In our results, we only need one subgraph to be connected, and therefore our work can be regarded as an extension of [27].

## Simulation example

In this section, we give two examples to illustrate the effectiveness of our results. In the first example, we consider the case with a fixed interaction topology and in the second example, we consider the case with a switching interaction topology. The robot model is taken as [28]

$$\begin{array}{ccc} & & \left(\begin{array}{cc} \theta_{1}+2\theta_{2}\cos\left(q_{i}\left(2\right)\right) & \theta_{3}+\theta_{2}\cos\left(q_{i}\left(2\right)\right)\\ \theta_{3}+\theta_{2}\cos\left(q_{i}\left(2\right)\right) & \theta_{3} \end{array}\right){\ddot{q}}_{i}\\ & & \;+\left(\begin{array}{cc} -2\theta_{2}\sin\left(q_{i}\left(2\right)\right){\dot{q}}_{i}\left(2\right) & -\theta_{2}\sin\left(q_{i}\left(2\right)\right){\dot{q}}_{i}\left(2\right)\\ \theta_{2}\sin\left(q_{i}\left(2\right)\right){\dot{q}}_{i}\left(1\right) & 0 \end{array}\right){\dot{q}}_{i}\\ & & \;+\left(\begin{array}{c} \theta_{4}\sin\left(q_{i}\left(1\right)\right)+\theta_{5}\sin\left(q_{i}\left(1\right)+q_{i}\left(2\right)\right)\\ \theta_{5}\sin\left(q_{i}\left(1\right)+q_{i}\left(2\right)\right) \end{array}\right)+\left(\begin{array}{c} u_{1}\\ u_{2} \end{array}\right)=\tau_{i} \end{array}$$ (67)

where $q_{i}={\left(q_{i}\left(1\right),q_{i}\left(2\right)\right)}^{T}\in R^{2}$ is the state vector and $u={\left(u_{1},u_{2}\right)}^{T}\in R^{2}$ is the uncertain term representing the summation of frictional vector and external disturbance. The model parameters are *θ*_1_ = 2.351, *θ*_2_ = 0.084, *θ*_3_ = 0.102, *θ*_4_ = 38.465, *θ*_5_ = 1.825. The desired trajectory for consensus tracking is

$$\begin{array}{c} q_{d}=\sin\left(0.1t\right)\left(\begin{array}{c} 1\\ 1 \end{array}\right). \end{array}$$

**Fig 2:**
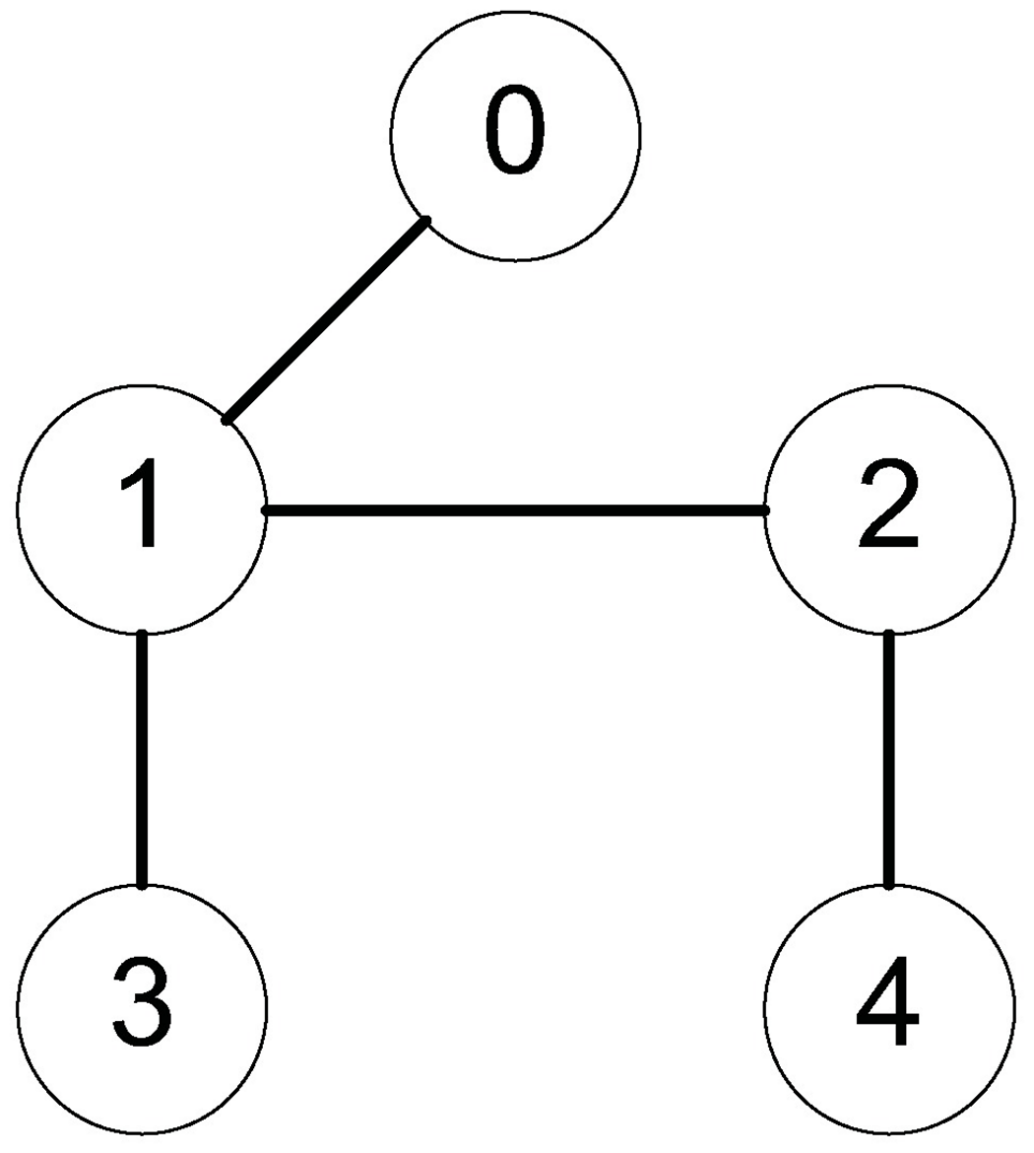
The fixed interaction topology.

**Fig 3:**
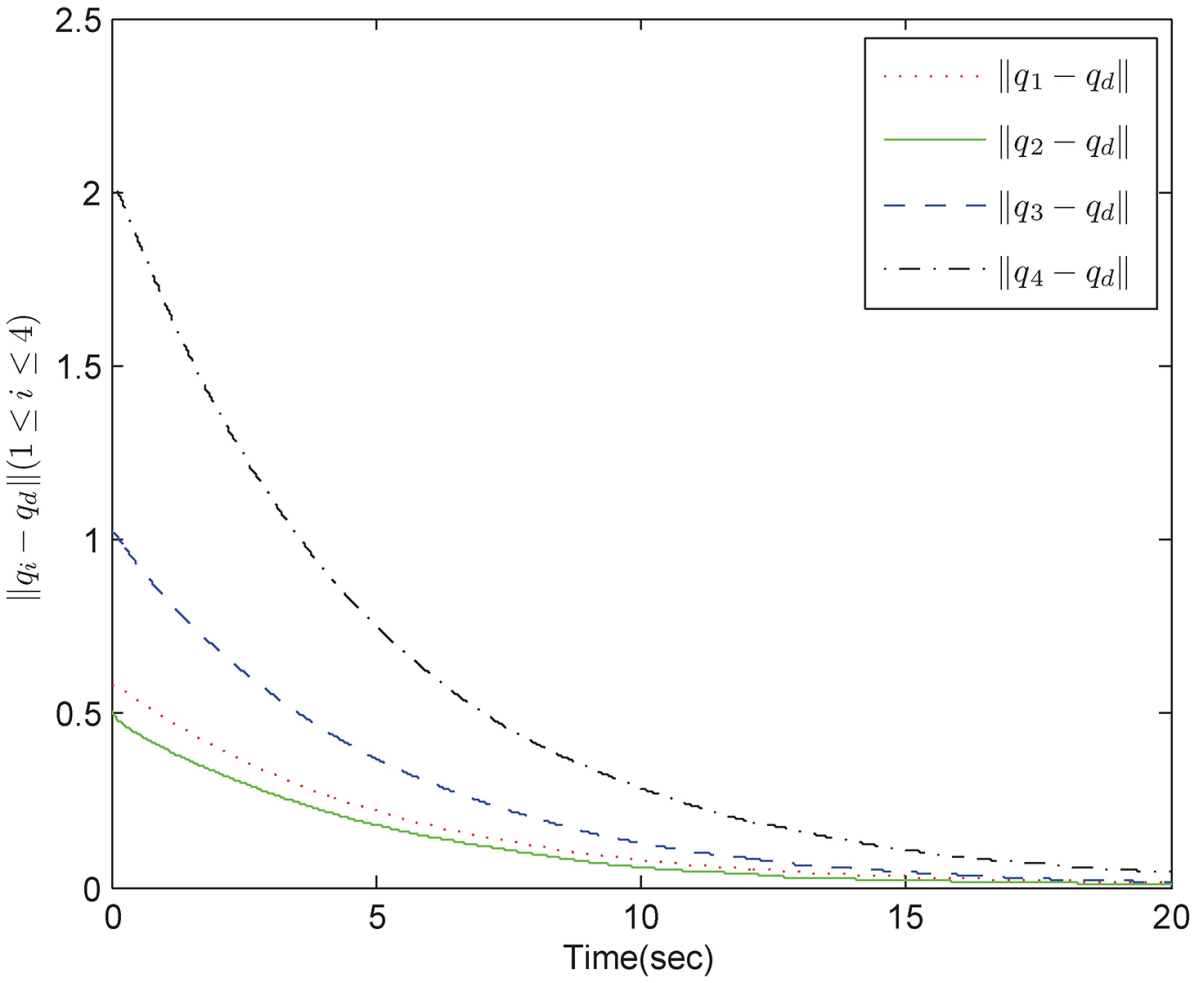
The position trajectory errors in fixed topology.

**Fig 4:**
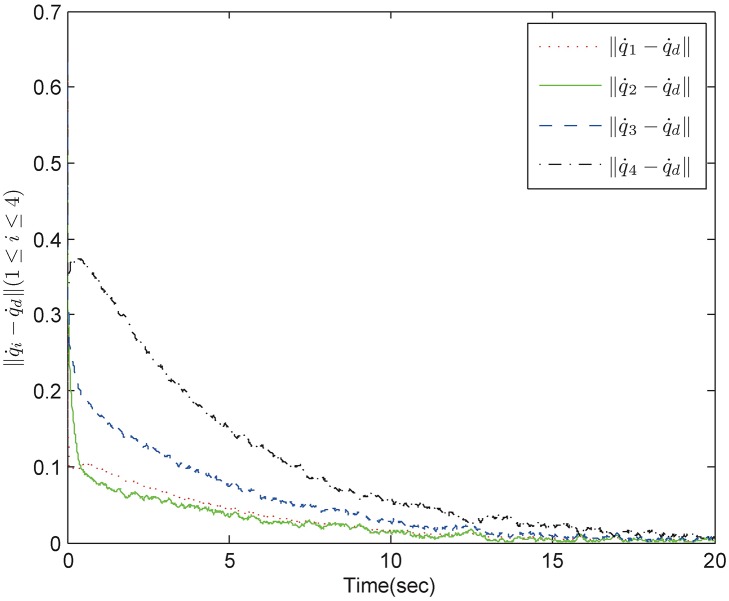
The velocity trajectory errors in fixed topology.

*Example 1:* Consider a group of mechanical systems consisting of four robots described as Eq (67). The interaction topology is shown in Fig 2. The initial conditions are chosen as $q_{1}={\left(0.5,-0.3\right)}^{T},q_{2}={\left(0.1,-0.5\right)}^{T},q_{3}={\left(0.2,-1\right)}^{T},q_{4}={\left(-0.4,2\right)}^{T},{\dot{q}}_{1}={\left(-0.1,0.7\right)}^{T},{\dot{q}}_{2}={\left(0.2,0.6\right)}^{T},{\dot{q}}_{3}={\left(0.7,-0.1\right)}^{T},{\dot{q}}_{4}={\left(0.4,-0.3\right)}^{T}.$ The bound of the uncertain terms is *ρ* = 0.1 and the sampling period is 0.01s. The control parameters for the robust tracking protocol are chosen as *α* = 5, *β* = 0.2, *ϵ* = 4 × 10^−4^. By using control inputs (10), (11), and (16), we obtain simulation results in Figs 3 and 4. Fig 3 shows the position errors of the multiple mechanical systems with frictional terms. It is apparent that the control scheme makes the systems converge to an agreement rapidly, which shows a good transient performance and final synchronization accuracy. Fig 4 shows the velocity errors of the multiple mechanical systems. As can be seen from the trajectories, the velocity errors converge fast to equilibrium points. According to the simulation results, we conclude that the proposed protocol can solve the robust tracking problem satisfactorily.

**Fig 5:**
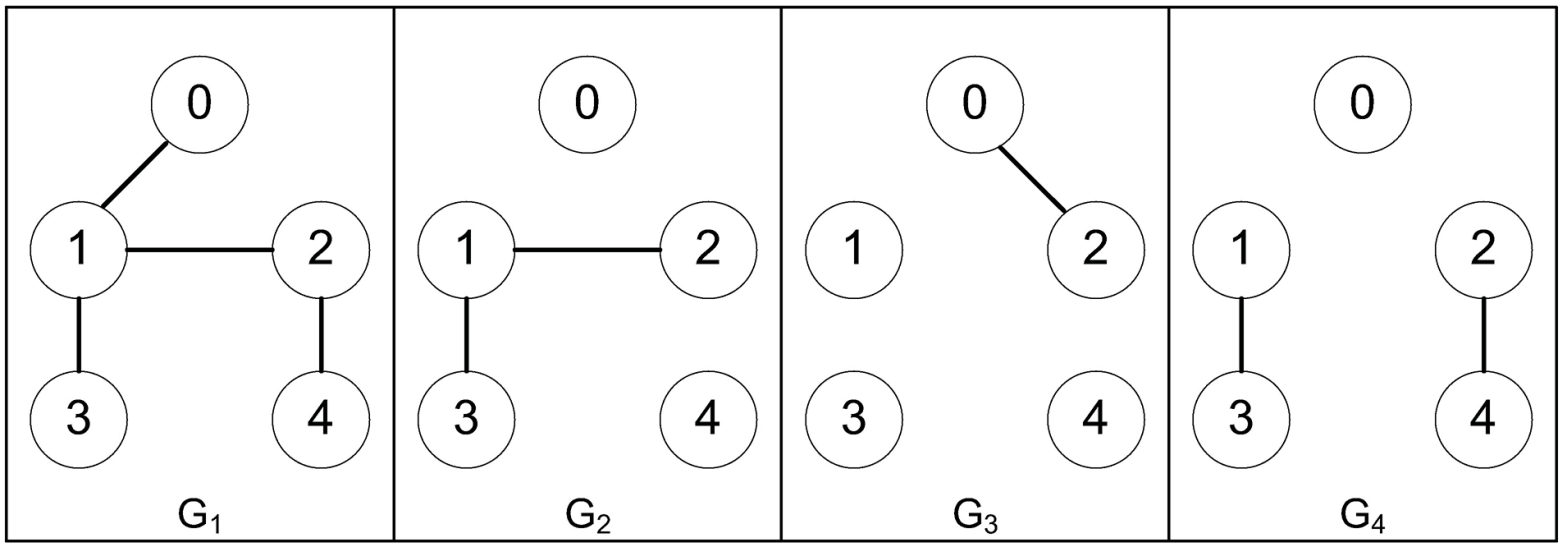
The switching interaction topologies.

**Fig 6:**
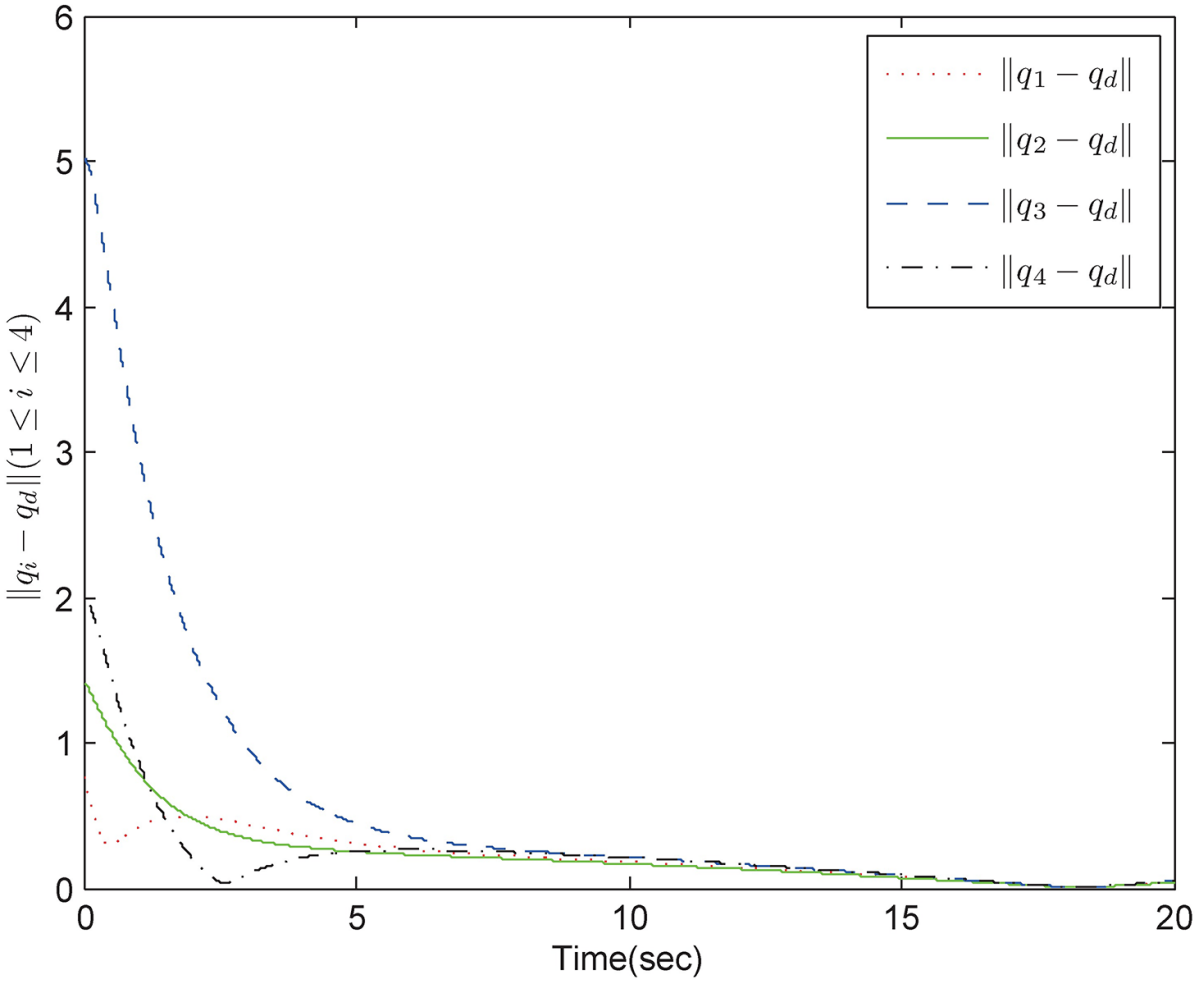
The position trajectory errors in switching topology.

**Fig 7:**
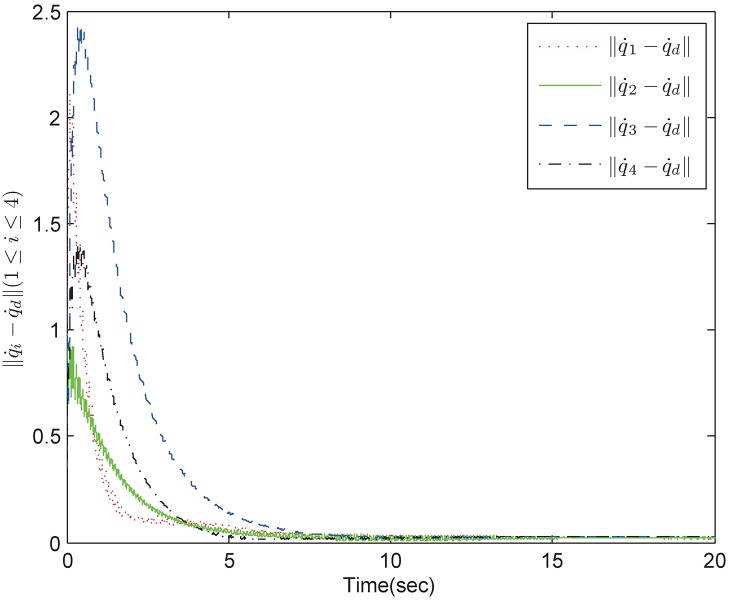
The velocity trajectory errors in switching topology.

*Example 2:* Consider a group of mechanical systems consisting of four robots described as Eq (67). The possible interaction topologies are {*G*_1_, *G*_2_, *G*_3_, *G*_4_} as shown in Fig 5. The interaction topologies are switched as *G*_1_ → *G*_2_ → *G*_3_ → *G*_4_ → *G*_1_ → …. Each topology is active for 0.025s and the sampling period is 1ms. The initial conditions are chosen as $q_{1}={\left(0.3,0.7\right)}^{T},q_{2}={\left(1,-1\right)}^{T},q_{3}={\left(0.2,-5\right)}^{T},q_{4}={\left(-0.4,2\right)}^{T},{\dot{q}}_{1}={\left(-0.5,0.2\right)}^{T},{\dot{q}}_{2}={\left(1,0.5\right)}^{T},{\dot{q}}_{3}={\left(0.6,-1\right)}^{T},{\dot{q}}_{4}={\left(0.2,-0.3\right)}^{T}$. The bound of the uncertain terms is *φ* = 0.04, and the control parameters for the robust tracking protocol are chosen as *μ* = 2, *d* = 0.5, *ϵ* = 10^−4^. The trajectory errors of each agent are shown in Figs 6 and 7. We can see that the system states converge to an agreement rapidly, which validate the effectiveness of the proposed robust consensus tracking protocol. The controller developed in this paper under switching topologies are different from existing results proposed in [27]. In [27], all the subgraphs *G*_*i*_, *i* = 1, 2, 3, 4, are assumed to be connected. However, in Fig 5, we can see that only subgraph *G*_1_ is assumed to be connected. Therefore, our results can be regarded as extensions of those in [27].

## Conclusion and future works

The robust tracking problem for a team of mechanical systems in the presence of friction forces and external disturbances is addressed in this paper. Distributed robust control laws are proposed for both fixed and switching interaction topologies such that the state of each agent converge to the desired trajectory. The control laws are designed based on computed torque approach. But this approach requires complicated computation for exact dynamical knowledge of physical parameters. Future works include the study of robust tracking problem in the condition that the physical parameters can not be computed precisely.
